# Shedding light on the composition of extreme microbial dark matter: alternative approaches for culturing extremophiles

**DOI:** 10.3389/fmicb.2023.1167718

**Published:** 2023-06-02

**Authors:** Júnia Schultz, Flúvio Modolon, Raquel Silva Peixoto, Alexandre Soares Rosado

**Affiliations:** ^1^Red Sea Research Center, King Abdullah University of Science and Technology, Thuwal, Saudi Arabia; ^2^Computational Bioscience Research Center, King Abdullah University of Science and Technology, Thuwal, Saudi Arabia; ^3^Laboratory of Molecular Microbial Ecology, Institute of Microbiology, Federal University of Rio de Janeiro, Rio de Janeiro, Brazil

**Keywords:** extremophiles, extreme environments, microbial cultivation, applied microbiology, culturomics

## Abstract

More than 20,000 species of prokaryotes (less than 1% of the estimated number of Earth’s microbial species) have been described thus far. However, the vast majority of microbes that inhabit extreme environments remain uncultured and this group is termed “microbial dark matter.” Little is known regarding the ecological functions and biotechnological potential of these underexplored extremophiles, thus representing a vast untapped and uncharacterized biological resource. Advances in microbial cultivation approaches are key for a detailed and comprehensive characterization of the roles of these microbes in shaping the environment and, ultimately, for their biotechnological exploitation, such as for extremophile-derived bioproducts (extremozymes, secondary metabolites, CRISPR Cas systems, and pigments, among others), astrobiology, and space exploration. Additional efforts to enhance culturable diversity are required due to the challenges imposed by extreme culturing and plating conditions. In this review, we summarize methods and technologies used to recover the microbial diversity of extreme environments, while discussing the advantages and disadvantages associated with each of these approaches. Additionally, this review describes alternative culturing strategies to retrieve novel taxa with their unknown genes, metabolisms, and ecological roles, with the ultimate goal of increasing the yields of more efficient bio-based products. This review thus summarizes the strategies used to unveil the hidden diversity of the microbiome of extreme environments and discusses the directions for future studies of microbial dark matter and its potential applications in biotechnology and astrobiology.

## 1. Introduction

Although extreme environments are complex and inhospitable, they often harbor a high microbial diversity (Shu and Huang, 2022). Extensive efforts have been made to fully characterize and describe novel extremophilic microbes and their metabolic functions in these harsh conditions using culture-dependent techniques (Vester et al., 2015; Topçuoglu and Holden, 2019; Sood et al., 2021; Schultz et al., 2022b). However, although there has been enormous progress in the isolation and culture of extremophiles, most of these microorganisms cannot be cultivated and fail to grow in conventional laboratory settings. Recent studies have estimated that 80% of microbial taxa remain uncultured (Lloyd et al., 2018) and 85% of the phylogenetic diversity of prokaryotes consists of yet-to-be-cultured taxa (Nayfach et al., 2021). Although there is no consensus regarding the percentage of the culturable fraction of prokaryotes and eukaryotes, most taxa, including extremophiles, clearly lack cultivated representatives. In turn, these yet-to-be-cultured taxa are termed “microbial dark matter” (Marcy et al., 2007; Rinke et al., 2013).

In view of the metabolic adaptations required for extremophiles to thrive in the severe conditions of extreme environments, studies on these organisms can expand our knowledge of the origin of life (on our planet and beyond), evolution, ecology, physiology, and biotechnology. However, the first step toward unlocking the full potential of extremophiles is to overcome the challenge of culturing them. In order to obtain pure cultures and fully understand the inadequately exploited cultured extremophiles, developing innovative culturing methods is crucial (Vartoukian et al., 2010; Bull and Goodfellow, 2019). Several old and recently developed techniques and strategies have been applied to isolate previously uncultured microbes from extreme habitats, such as prolonging incubation times (Davis et al., 2005; de Jesus et al., 2015; Pulschen et al., 2017), using different concentrations of oxygen and other gases (Lopez et al., 2019; Volpiano et al., 2021), using low-nutrient culture media (Do Carmo et al., 2011; Peixoto et al., 2011; Grzesiak et al., 2015; Pulschen et al., 2017), adding antibiotics to inhibit fast-growing microorganisms and prevent contamination with unwanted microbial groups (Bender et al., 2020), changing the gelling agent, e.g., gellan gum (Das et al., 2015), or using a cellulose plate (Tsudome et al., 2009) instead of agar, *in situ* diffusion devices (Nichols et al., 2010; Palma Esposito et al., 2018), and cell-targeting methods (Huber et al., 2000; Antunes et al., 2008a). However, although these alternative strategies may yield new microbes, most of the microbes presumed to occur in extreme environments remain uncultured.

**Figure 1:**
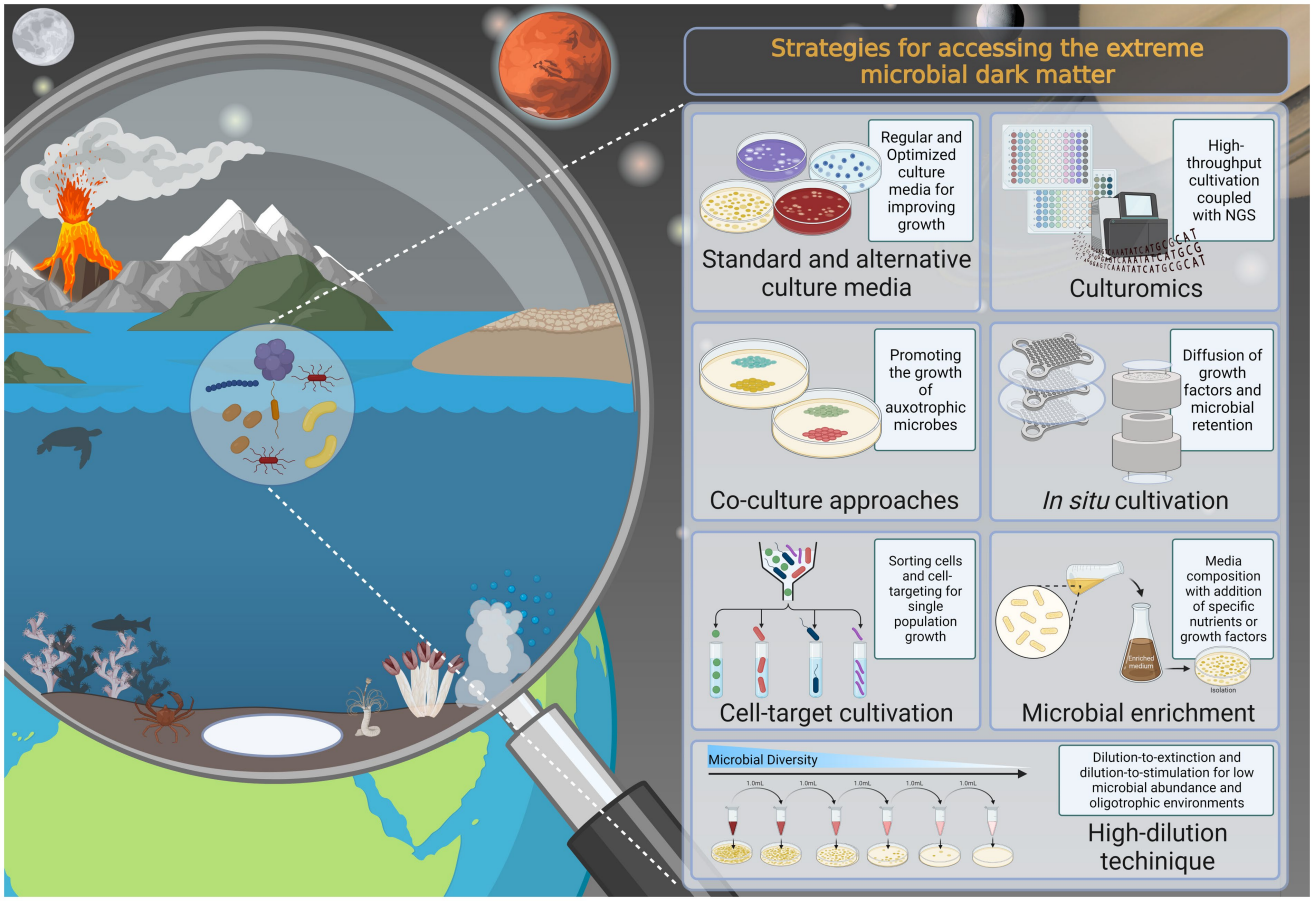
Overview of the current knowledge of standard and alternative culture-dependent approaches that can be applied for culturing microbiomes from extreme environments and unveiling the members of the extreme microbial dark matter, in addition to enabling the culture of uncultivated extremophiles. The figure was created by the authors using Biorender.com.

This review summarizes methods and technologies used to recover the microbial diversity of extreme environments (often involving multiple extreme conditions; Figure 1) and discusses the advantages and disadvantages of each of these approaches (see Table 1). Additionally, we describe alternative culturing strategies to retrieve novel taxa and reveal previously unknown genes, metabolic processes, ecological roles, and more efficient bio-based products. Therefore, this study provides a comprehensive synthesis of the strategies used to unveil the hidden microbial diversity in extreme environments, while also suggesting further directions and improvements to make new discoveries and shed light on the microbial dark matter thriving in extreme environments.

**Table 1:** Summary of the main strategies to improve the culturability and isolation of extremophilic microorganisms from extreme environments.

Strategy	Approach	Microbial target	Previous uses	Advantages	Disadvantages	References
Culture media and incubation settings for microbial growth	Standard and alternative culture media, modifications of composition and incubation conditions	Generalist and specialized microorganisms	Environmental and clinical microbes. Commonly used for cultivation of microbes from extreme environments	-Easy formulation and manipulation	Cultivation bias of isolating more members of Proteobacteria, Firmicutes, Bacteroidetes and Actinobacteria	Landreau et al. (2016); Kurm et al. (2019); Pulschen et al. (2018); Yasir et al. (2019); Bender et al. (2020); Gómez-Acata et al. (2021); Molina-Menor et al. (2021)
				-Established methods and commercial options		
				-Low cost		
				-The equipment is not necessarily complex		
	Enriched culture	Generalist and specialized microorganisms	Environmental and clinical microbes. Commonly used for Archaea	-Enrichment of wanted groups by manipulating the conditions (e.g., adding inhibitors or growth factors)	-Long incubation period	Bae et al. (2005); Könneke et al. (2005); Klein et al. (2022)
				-Separation of specific species from a mixed community	-In some cases, lack of pure cultures	
	Co-culture	Autotrophic and syntrophic microbes	Environmental and clinical microbes	-Microbial interactions	-May require optimization for some requirements	Plugge and Stams (2002); Stewart et al. (2012); Sánchez-Andrea et al. (2018); Pillot et al. (2020)
				-Growth of microbes that depend on specific (sometimes unknown) compounds generated by other microorganisms	-Lack of pure cultures	
				-Low cost		
	Culturomics	Generalist and selective microorganisms	Environmental microbes and human gut microbiota	-High-throughput isolation	-Labor-intensive. Large numbers of samples and data to process	Lagier et al. (2012); Khelaifia et al. (2018); Yasir et al. (2019); Sood et al. (2021)
				-Multiple and simultaneous microbial growth under different conditions	-Significant cost	
Diffusion-based devices and *in-situ* cultivation	Cultivation chambers	Generalist microbes	Microorganisms from soil, marine environments, activated sludge	-*In situ* cultivation	Competition between cells may occur in the chamber, leading to selectivity	Kaeberlein et al. (2002); Bollmann and Lewis (2007)
				-Small, low-cost device		
	Isolation chips	Generalist microbes	Environmental microbes	-High-throughput cultivation	Difficulty of loading cells into the chip wells	Nichols et al. (2010); Berdy et al. (2017)
				-*In-situ* cultivation		
				-Easy observation of colonies under a microscope		
	Multiwell microbial culture chip	Generalist microbes	Environmental microbes	-High cultivation efficiency	Difficult to pick and recover microcolonies from the wells	Ingham et al. (2007)
				- High-throughput screening for phenotypes and microbial products		
				-Rapid changes in environment and analysis under a variety of culture conditions		
	Hollow-fiber membrane-based chamber	Generalist and specialized microorganisms	Environmental microbes	-Simple handling	-Oversize device	Aoi et al. (2009); Fu et al. (2017)
				-Rapid molecular exchange	-Culture of microbes from surface water layer	
	Paper-based analytical device	Generalist microbes	Environmental, human and clinical microbes	-Multifunctional, used for biological and chemical purposes	Selective growth of well-known cultured microbes	Kim and Yeo (2016); Noiphung and Laiwattanapaisal (2019); Levy et al. (2020)
				-Low cost		
				-Efficient for clinical diagnostics		
Targeted cell-sorting and cultivation	Reverse genomics	Targeted microbial groups	Human oral microbiome	Isolation and cultivation of targeted microbes	-Significant cost	Cross et al. (2019); Ibrahim et al. (2022)
					-Use of different robust equipment	
					-Required technical abilities	
	FACS	Targeted microbial groups	Environmental and clinical microbes	Isolation and cultivation of targeted microbes	-Not compatible for anaerobe growth	Alvarez-Barrientos et al. (2000); Zengler et al. (2002); Espina et al. (2022)
					-In some cases, it is not possible to culture	
	Live-FISH	Targeted microbial groups	Marine microbes	-Isolation and cultivation of targeted microbes	-Advances skills in fluorescence due to variations in fluorescent signals can decrease the detectability of some microorganisms	Batani et al. (2019)
				-No sophisticated probe design is required		
	Micromanipulators and laser manipulation system	Generalist and selective microorganisms	Marine microbes, human cells, microorganisms from deep-sea brine pools and hydrothermal systems	-Selection of cells of interest from a mixed microbial community	-Required technical abilities	Huber et al. (1995); Huber et al. (2000); Antunes et al. (2008a,b)
				-Pure colonies obtained by sorting cells with microscope	-Laser manipulation	

## 2. Strategies to access extreme microbiomes

Culture-dependent and -independent methods are widely used to gain insights into the ecology, physiology, microbial interactions and dynamics, and functional roles of the culturable fraction of microbiome members. These techniques mainly use standardized methods, with nutrient-rich culture media and standard pH, salinity, oxygen, and temperature parameters. Alternative approaches have been used in an attempt to cultivate hard-to-culture microbes by varying the cultivation settings (Pulschen et al., 2017; Lagier et al., 2018), *in situ* cultivation, using diffusion-based devices (Steinert et al., 2014; Berdy et al., 2017), and a combination of the aforementioned technologies.

Cultivation-independent techniques enable the study of the overall function and activity of microbial communities in the environment and have allowed for the identification of novel bacterial groups and the assessment of the biotechnological potential of microbial communities by identifying useful pathways and genes (Sysoev et al., 2021). The number of known taxa can also be further expanded by omitting culture steps. Zamkovaya et al. (2021) recently demonstrated that the members of the microbial “dark matter” from different environments (including extremes) play key roles in ecological networking within their respective communities.

Recently, Shu and Huang (2022) made a substantial effort to summarize the current state of the characterization of prokaryotes inhabiting the major extreme environments on Earth. Data from culture-independent methods provided insights regarding the microbial composition and diversity of complex environments. For instance, hot springs (water and sediment) are dominated by the phyla Aquificae, Proteobacteria, and Crenarchaeota, whereas Proteobacteria is the dominant phylum in the deep sea and the archaeal phylum Crenarchaeota is the most abundant in hypersaline habitats. Prokaryotes in the cryosphere (glaciers and permafrost) are diverse, particularly the phyla Proteobacteria, Chloroflexi, and Actinobacteria. Diversity surveys using marker genes and genome-resolved metagenomics have unveiled a vast range of previously unknown microorganisms in extreme environments, thus substantially expanding the phylogenetic breadth and genome representation of the tree of life (Hug et al., 2016; Shu and Huang, 2022). However, the understanding of their physiology and ecology is hampered by the lack of pure cultures (Hedlund et al., 2015). The lack of cultured representatives of many of the known microbial taxa reinforces the need for more efficient tools to culture these organisms, especially given that many play key functional roles in communities (e.g., biogeochemical cycles) in extreme environments and are important to explain the origin and evolutionary processes of microorganisms (Merino et al., 2019; Martínez-Espinosa, 2020). Here, we describe the factors that strongly influence the culturability of extremophiles in laboratory conditions, highlighting the main techniques and strategies used to culture these microbes.

### 2.1. Sample processing

Sample processing interferes with the culturability of the microbial community from any environment. This problem can be even more challenging for samples from extreme environments. The first step to successfully culture extremophiles, as with any other microbe (Bellali et al., 2019; Schultz et al., 2022a), is to effectively detach the microbial cells from the sample substrate. Using three different resuspension buffers (Ringer’s solution, PBS, and sterile cave water, pH 8.2) for rocks from cave samples, Bender et al. (2020) identified the largest number of colonies and diverse phenotypes in water. This suggests that suspension solutions buffered to physiological conditions (i.e., pH, sodium, and potassium) should be effective in increasing microbial culturability (Padan et al., 2005). For extremophiles, resuspending samples in sterilized water obtained from a given environment or suspensions using a buffer that is geochemically similar to the environment could be the best option, as osmotic stress and low salinity may induce a viable but non-culturable state in bacteria (Gin and Goh, 2013; Li et al., 2014; Dong et al., 2020). Sprinkling portions of the sample onto Petri dishes containing culture media (e.g., R2A and MA media) could be an alternative to resuspension and could eventually increase the recovery of different and more-pigmented microbial morphotypes compared to classical serial dilution, as reported by Paulino-Lima et al. (2016), who cultured bacteria from sand samples collected from the Atacama Desert.

Extreme environments are usually remote, difficult to access, and far from the laboratory. Processing time and primary incubation after sampling also directly impact culturability. Bender et al. (2020) evaluated the significance of crushing and plating the samples immediately after sampling versus 6 h later. When sample processing was delayed, the number of colonies and their diversity were sharply reduced (Bender et al., 2020). Similarly, Bellali et al. (2019) described the negative impact of delaying the processing and first cultivation of gut microbiota, as the viability and diversity of microbes were significantly reduced when sample processing was delayed for increasingly long periods.

### 2.2. Moving forward: advances in microbial cultivation

In the early 1980s, Staley and Konopka described the discrepancy between the total number of microbial cells in an environmental sample and the culturable population of that sample as the “great plate count anomaly.” Only a small fraction of the true microbial diversity and cell abundance in any environment can be retrieved by culturing (Nichols et al., 2008), whereas the remaining microbes are recalcitrant to cultivation (Joint et al., 2010). Classical methods include using nutrient-rich or nutrient-poor culture media coupled with standard incubation times, pH, salinity, oxygen, and temperature (Pulschen et al., 2017; Schultz et al., 2022a). However, these methods appear to work best for fast-growing organisms, which represent only a small fraction of natural microbial communities (Pham and Kim, 2012; Sysoev et al., 2021).

Cultivation-based tools are still paramount to achieve a comprehensive understanding of the biology, ecology, and bioactivity of extremophiles, and are essential for future bioprospecting and other applications (Joint et al., 2010). The value of culturing the extremosphere microbiome is undeniable. For example, cultivation has enabled the discovery of several widely used enzymes (Chien et al., 1976; Mesbah, 2022) and has provided valuable insights into the origin and evolution of life on Earth (Merino et al., 2019; Thombre et al., 2020), in addition to providing a means to evaluate the prospect of extraterrestrial life, planetary protection, and space exploration (Cavicchioli, 2002; Carré et al., 2022; Blachowicz et al., 2022). For this reason, there is a growing interest in culturing and recovering a larger proportion of extremophilic microorganisms from a sample, and this can be achieved by improving cultivation methods. The use of alternative approaches such as different cultivation settings (Song et al., 2009), the formulation of new culture media (Tyson et al., 2005; Ñancucheo et al., 2016), complex microfluid and laser manipulation systems (Imachi et al., 2011), filters and membrane systems to simulate the natural environment (Ferrari et al., 2008; Benaud et al., 2022), co-culture approaches (Nichols et al., 2008; Shaw et al., 2020), and *in situ* cultivation using diffusion-based devices (Kaeberlein et al., 2002; Nichols et al., 2010; Pope et al., 2022) could overcome challenges in mimicking the natural environment and isolating hard-to-culture microbes (Figure 1; Table 1).

#### 2.2.1. All-you-can-eat: standard and alternative culture media and growth conditions

To effectively cultivate a given microorganism, its physiological and metabolic needs must first be addressed (Leadbetter, 2003). These factors are especially important in feeding extremophiles. An all-you-can-eat buffet of amino acids and sugars, such as those found in standard media formulations, is not necessarily the best approach. The most basic approach used to culture microbes from different sources (e.g., extreme environments) is the use of conventional nutrient-rich culture media (e.g., tryptic soy broth, lysogen broth, marine broth, nutrient broth, etc.). New materials may revolutionize microbiology by improving access to organisms that were previously recalcitrant to culture. The design of alternative media may increase the number of different microorganisms, including modification of culture medium composition, the addition of specific chemicals and promoters, depleting carbon sources, and altering the gelling agent. However, this requires in-depth insights into the nature and characteristics of the samples to better understand the factors that determine bacterial growth (Pham and Kim, 2012).

Hamaki et al. (2005) developed an innovative soil-extract agar medium in an effort to mimic the characteristics of the soil environment and cultivate soil microbes. This newly developed synthetic medium enabled the growth of novel bacteria (Actinomycetes) due to the presence of soil constituents that are required for the growth of some groups of microorganisms. Gómez-Acata et al. (2021) used a soil-extract agar medium to recover 37 haloalkaliphilic strains from volcanic samples. Most of the samples collected in extreme environments have a low level of organic carbon. Excessive amounts of carbon in conventional culture media can interfere with microbial growth, and therefore reducing the organic-carbon input might be an effective strategy for bacterial culture, particularly for oligotrophic taxa (Kurm et al., 2019; Bender et al., 2020). The same is true for nutrient concentrations (Reasoner and Geldreich, 1985). Extreme environments tend to be nutrient-poor, and the large amounts of nutrients in rich culture media can induce a high-nutrient shock and impair the growth of microorganisms that inhabit stressful low-nutrient environments. This problem led to the development of the low-nutrient R2A culture medium, which is extensively used in cultivating extremophiles (Reasoner and Geldreich, 1985; Bendia et al., 2018; Kusuma et al., 2022). Using low-nutrient media (usually diluting standard culture media), Pulschen et al. (2017) and Molina-Menor et al. (2021) were able to retrieve microorganisms belonging to rare or recently described genera and to cultivate previously uncultured Antarctic and European desert bacteria, respectively.

The gelling agent is also an important but often overlooked factor that can strongly influence microbial culturability, and is a key driver of the structure of different cultured communities (Mac Rygaard et al., 2017). Agar is the most commonly used gelling agent. However, there are other compounds with similar gelling properties, including xanthan gum, carrageenan, isubgol, and gellan gum. These polymers can be used to culture organisms that grow poorly or not at all on agar and may increase their growth rates (Das et al., 2015). Agar is derived from a group of red marine algae (genera *Gelidium* and *Gracilaria*) and has been used in microbiology since 1882 (Hitchens and Leikind, 1939). Gellan gum is an extracellular polysaccharide secreted by the bacterium *Sphingomonas elodea*. This compound is commercially manufactured by a fermentation process and possesses unique colloidal and gelling properties, a good ability to form coatings, and high clarity (Das et al., 2015). Due to its high thermal stability, gellan gum has been used to culture (hyper)thermophiles (Shungu et al., 1983; Landreau et al., 2016). Several studies have reported that gellan gum improves the culturability of certain microorganisms. For instance, Sari et al. (2020) cultivated rare thermophilic Actinobacteria; Landreau et al. (2016) cultured several anaerobic (hyper)thermophilic marine microbes; and Ferrari et al. (2008) isolated and studied previously unculturable bacteria from soil using an alternative gelling gum. Regarding acidophiles, Johnson (1995) found that agarose or gellan gum must be used instead of agar, as agar is prone to hydrolysis in low pH conditions. Moreover, the gelling agent must be sterilized separately and later combined with the acidic medium components. Johnson (1995) also found that culturing of heterotrophic acidophiles is improved when they are grown in a double-layer overlay solid medium. The use of polycarbonate filters floating on an acidic liquid medium containing ferrous sulfate enables the growth of acidophiles on a solid medium (de Bruyn et al., 1990). These examples demonstrate the importance of selecting the optimal gelling agent for a particular purpose, especially when culturing extremophiles. In another example, Tsudome et al. (2009) described the advantages of a matrix made of nanofibrous cellulose instead of agar, which is more stable, even at temperatures exceeding 100°C.

In an attempt to grow unculturable microbes, microbiologists also often change the growth conditions, such as the incubation period, inoculum size, temperature, pH, and atmosphere (CO_2_/O_2_ level). By applying a combination of simple techniques such as low temperatures (12°C) and a long incubation period (up to 15 weeks), Pulschen et al. (2017) increased the culturable diversity and recovered previously uncultured microorganisms, thus revealing a rare bacterial diversity in a soil sample from Antarctica. Additionally, the plates were incubated in the dark and in polyethylene bags to prevent drying. Similarly, prolonged incubation (3 months) was crucial for the growth of rarely isolated phyla of soil microbes (Davis et al., 2005; Steven et al., 2007). Burns et al. (2004) obtained an increased diversity of Haloarchaea with incubation times as long as 12 weeks. Extended incubation times (more than 8 months) yielded the highest diversity of cultured bacteria and unknown species from a cold and alkaline environment (Vester et al., 2013).

Different culture-based strategies can be applied for slow-growing bacteria, especially due to the bias toward the cultivation of fast-growing bacteria. For instance, recovery of slower-growing species can be improved by adding low concentrations of antibiotics (e.g., chloramphenicol, amphotericin B, and nalidixic acid), which will slow down or completely inhibit the growth of opportunistic fast-growing species with flexible metabolisms (Alain and Querellou, 2009). Weissman et al. (2021) compared the growth rates of cultivated and uncultivated organisms to illustrate how culture collections are strongly biased toward organisms capable of rapid growth. The authors found that organisms naturally group into two growth classes and observed a bias in growth predictions for extremely slow-growing organisms. In an effort to recover slow-growing bacteria from hot springs, Yasir et al. (2019) used low-nutrient media supplemented with water from each respective hot spring. The authors also added amphotericin B to the medium to prevent fungal contamination and supplemented it with ascorbic acid to support the growth of anaerobic bacteria in an aerobic environment. With these adaptations, 536 strains affiliated with 139 distinct species were isolated after 15 days of incubation. From cave samples, Bender et al. (2020) evaluated the effect of antibiotics on culturability, preparing media with and without 10 μg/mL chloramphenicol and nalidixic acid, and observed inhibition of the growth of rapidly growing species and a statistically significant increase in colony counts. For the cultivation of extreme halophiles, Robinson et al. (2005) obtained higher diversity when the solid medium was supplemented with penicillin.

A novel strategy termed “selective medium-design algorithm restricted by two constraints” (SMART) was developed by Kawanishi et al. (2011). This method for the culture of a target microorganism from a complex environment is based on two selective agents: (i) a carbon source, enabling proliferation of the target microorganism, and (ii) antimicrobials, suppressing unwanted microorganisms in the medium. The authors were able to successfully grow only the intended microbes and effectively suppress the growth of unwanted microorganisms. Likewise, the SMART approach allowed for the isolation of the fish pathogen *Edwardsiella tarda*, which was inoculated into a culture medium containing a mixture of other fish pathogens (Yamamoto et al., 2017). However, additional tests using the SMART approach in microbial cultures from extreme environments are needed to confirm the potential of this alternative method to culture extremophiles.

#### 2.2.2. Enrichment as a strategy to increase culture diversity

In microbial cultivation, enriched media are commonly used to facilitate the growth of certain microbes with specific metabolic requirements that are not supplied (and/or could be inhibited) by conventional culture methods. One example is the cultivation of archaeons, which commonly requires extreme conditions, and the enrichment step must use specific molecules to mimic environmental conditions. These requirements include physical and chemical conditions besides growth factors, such as energy and nutrient sources (Sun et al., 2020). For instance, archaeons from the phyla Thaumarchaeota and Crenarchaeota have been successfully grown by using ammonia as an electron donor (Könneke et al., 2005), as they oxidize ammonia to nitrite as a primary energy source.

Enrichment is another strategy that has also allowed for the culture of Asgardarchaeal members. Imachi et al. (2020) have succeeded in culturing an Asgard archaeon, *Candidatus* Prometheoarchaeum syntrophicum strain MK-D1, from deep marine sediment. This strain was characterized as a syntrophy between either a methanogenic archaeon, a sulfate-reducing deltaproteobacterium, or both. Moreover, the authors reported that the archaeon grows very slowly, taking over a decade for enrichment. The cultivation of *Ca. P. syntrophicum* was crucial to rest criticisms regarding their existence since their first discovery from binning metagenome-assembled genomes (MAGs; López-García and Moreira, 2020). Later, Rodrigues-Oliveira et al. (2023) reported a highly enriched culture of *Candidatus* Lokiarchaeum ossiferum, another member of the Asgard phylum. This species grew anaerobically at 20°C in the presence of organic carbon sources and exhibited a significantly larger genome compared with the single previously cultivated Asgard strain (Imachi et al., 2020). Additionally, enrichment is a great strategy to culture methane- and alkane-oxidizing microbial consortia. To achieve this, a sample of the target environment (e.g., soil, sediment, or water) is collected and then placed in a suitable growth medium that contains methane or alkane as the sole source of carbon and energy, after which the media is adjusted to the required temperature. Over time, the microbial community will adapt to the new conditions, and the methane- and alkane-oxidizing consortia will enrich and become more abundant (Holler et al., 2011; Dowell et al., 2016). For example, Holler et al. (2011) were able to enrich a consortium of anaerobic methane-oxidizing archaea of the ANME-1 lineage consortia and the deltaproteobacterial HotSeep-1 cluster, which were thriving under thermophilic enrichment conditions.

Unknown growth factors could be supplemented using raw extracts from the original source (Schultz et al., 2022a). Some microbes use cell components of other microbes as growth factors and microbial extracts could enhance the cultivation of these extremophiles (Bae et al., 2005). However, depending on the nature of the sample, the extruded components can be toxic to some microbes after extraction or sterilization. Overgrowth of other (unwanted) microbes such as generalist bacteria or fungi may also inhibit the growth of archaea. Extremophile microorganisms generally exhibit slow growth rates and cannot be easily differentiated based on colony morphology. Additionally, these microorganisms are often inhibited by competition or by specific components of a culture medium (Sun et al., 2020; Klein et al., 2022). To inhibit the overgrowth of such unwanted microbes, antibiotics could be added to the culture medium (see topic 2.2.1). Once the enrichment steps are successfully carried out, isolation could be performed in some cases.

#### 2.2.3. High-dilution techniques

High-dilution strategies are commonly used for culturing microorganisms from very low abundance samples, including soil, seawater, or other environmental samples. The basic principle of high-dilution culturing is to serially dilute the given sample until individual cells or colonies can be isolated from separate cultures (Button et al., 1993; Connon and Giovannoni, 2002). This technique is useful for studying microbial diversity and physiology in environments with low microbial abundance.

In seawater, for instance, oligotrophic and ultra-oligotrophic prokaryotes are typically found in very low abundance, making them difficult to study. However, high-dilution approaches have been used to successfully cultivate these organisms (Rappé et al., 2002; Cho and Giovannoni, 2004; Henson et al., 2016). The “dilution-to-extinction” method involves serially diluting mixed culture samples to the point where individual cells are isolated (Button et al., 1993). This technique has been used to cultivate a wide range of oligotrophic bacteria and archaea from seawater and later utilized to cultivate groundwater (Connon and Giovannoni, 2002) and lake water (Salcher et al., 2015) bacterioplankton. Using this approach, previously uncultured marine oligotrophic bacterioplankton, including the predominant SAR11 clade (*Candidatus* Pelagibacter; Rappé et al., 2002; Song et al., 2009), the SAR 116 clade (*Candidatus* Puniceispirillum; Henson et al., 2016), the OM43 clade (Giovannoni et al., 2008), the OM60 clade (Cho et al., 2007), the SUP05 clade (Spietz et al., 2019), and the oligotrophic marine Gammaproteobacteria group (Cho and Giovannoni, 2004), have been successfully cultivated in a laboratory setting and their physiologies and genomes have been successfully investigated. Additionally, the “dilution-to-extinction” technique has been used for the isolation of ammonia-oxidizing bacteria (e.g., *Nitrosospira* spp. and *Nitrosomonas* spp.) from soil samples (Aakra et al., 1999), as well as for the isolation of thermophilic piezophiles (Kato, 2011).

The “dilution-to-stimulation” method is another strategy that involves adding a small amount of nutrients to seawater samples before diluting them (Ho et al., 2012). This approach has been used to successfully cultivate ultra-oligotrophic bacteria, which require extremely low levels of nutrients to grow, as well as to isolate a functional consortium for the production of hydrogen from cellulosic feedstocks (Ho et al., 2012). Another example was the experiment developed by Díaz-García et al. (2021), in which the authors presented a strategy to assemble a minimal and effective lignocellulolytic bacterial consortium where the first step was the “dilution-to-stimulation” approach. From the “dilution-to-stimulation” phase, several bacterial types were significantly enriched, including members of the Paenibacillaceae, Sphingobacteriaceae, Enterobacteriaceae, and Pseudomonadaceae families. Both of these high-dilution approaches require careful attention to sterile technique and appropriate culturing conditions, as well as time and persistence. Nevertheless, they have proven to be valuable tools for exploring the diversity and physiology of oligotrophic microorganisms and functional microbial consortia from environmental samples.

#### 2.2.4. Syntrophy

Another limitation in extremophile cultivation is the syntrophic metabolism of some microbes. In other words, microbes may lack some metabolic apparatus and supplement it by feeding on metabolites produced by other microbes. In the natural environment, most microorganisms grow in a consortium with other microbes of the same or different taxa. In general, syntrophy allows a consortium of microorganisms to gain energy by coupling processes that can, for bioenergetic reasons, be accomplished only through microbial interlinkage (Moissl-Eichinger et al., 2018). Cooperative interaction among microbes is an important survival strategy (Mee et al., 2014). Mimicking complex networks that naturally occur in extreme environments can also provide insights into the limitations of the traditional cultivation of individual microorganisms (Marmann et al., 2014), in which microbes can cooperate through the exchange of metabolites and signaling molecules within a shared pool of micronutrients (Pande and Kost, 2017). Thus, coculturing enables obligate symbionts to grow with their microbial partners and may activate genes that are not expressed in laboratory settings and in pure cultures, thus improving culturing success (Bertrand et al., 2014). For example, the coculture approach successfully enabled the culture of *Candidatus* Prometheoarchaeum syntrophicum strain MK-D1, a member of the Asgardarchaeota (proposed superphylum) cultured from deep marine sediments (for more information, see section 2.2.2).

Extremophiles are uniquely difficult to investigate due to their complex syntrophy. Nevertheless, the results derived from these studies are often fascinating. For example, by using the coculture strategy, Sánchez-Andrea et al. (2018) successfully enhanced sulfidogenesis by adding the sulfur reducer *Desulfurella amilsii* to a culture of the acidophilic fermentative bacterium *Lucifera butyrica.* Similarly, Pillot et al. (2020) reported the generation of electricity through the syntrophy between exoelectrogenic and fermentative hyperthermophilic microbes from hydrothermal vents. These experiments demonstrated the direct production of electric current from acetate, pyruvate, and H_2_ and indirect production from yeast extract and peptone through the production of H_2_ and acetate from fermentation. Methanogenesis is a well-studied process performed by syntrophic microbes (Sieber et al., 2010; Phan et al., 2021; Tilahun et al., 2021). Syntrophic acetate oxidation is catalyzed by syntrophic acetate-oxidizing bacteria, whereas H_2_-consuming methanogenesis is catalyzed by hydrogenotrophic methanogens. The two microbes obligately require each other, since the bacterium requires hydrogen scavengers (i.e., partner methanogens) and the archaeon requires hydrogen suppliers (i.e., syntrophic acetate-oxidizing bacteria). Although this mutual syntrophy theoretically yields energy, the amount is quite small (ΔG0’ = 31.0 kJ/mol). Moreover, the syntrophic acetate-oxidizer and partner methanogens must share this small amount of energy. This energy disadvantage may cause these syntrophs to grow slowly and adopt a rigid mutualism, which may explain why the isolation of syntrophic acetate-oxidizing co-cultures was long considered extremely difficult or even impossible (Hattori, 2008).

#### 2.2.5. *In situ* diffusion-based devices in extreme environments

Platforms for *in situ* cultivation that were successfully developed in the last decade have improved the cultivation of certain microbial groups that were previously difficult or impossible to culture. These improvements were made based on observations of the natural environment, which provides all of the initiation factors required for microbial growth (Jung et al., 2021). These platforms could be implemented as devices customized for different environments and specific purposes. Some examples of applications include the cultivation of microbes from wet soil (Ling et al., 2015; Berdy et al., 2017; Chaudhary et al., 2019), marine systems (Aoi et al., 2009; Alkayyali et al., 2021), and different hosts such as sponges (Steinert et al., 2014; Jung et al., 2021) and corals (Schultz et al., 2022b).

Chaudhary and Kim (2019) developed a diffusion bioreactor that consisted of a container wrapped with a polycarbonate membrane (0.4 μm) filled with culture media, which was then placed in the soil. Similar to other *in situ* diffusion-based devices, the polycarbonate membrane allowed for the exchange of essential compounds with the environment (nutrients and oxygen, for example), thus enabling the culture of soil microbes such as members of the Proteobacteria, Firmicutes, Actinobacteria, and Bacteroidetes phyla (Chaudhary et al., 2019). Multiwell microbial culture chips are another alternative tool for microbial culturing, which were first used in freshwater by Ingham et al. (2007). The authors successfully conducted a high-throughput screening and their proposed approach enabled the rapid growth of novel bacterial species with potential for biotechnology (Ingham et al., 2007). Likewise, Palma Esposito et al. (2018) designed a miniaturized culture chip (microscreen plate), which was then applied to culture microorganisms from Antarctic sediments. Using this approach, the authors were able to isolate *Aequorivita* sp., a rare species with antimicrobial and anthelmintic activities. Based on the same concept of *in situ* incubation, Zengler et al. (2005) added single cells into microcapsules and exposed them to a continuous flow of culture medium under *in vitro* conditions, which enabled the retrieval of nearly 10,000 microcolonies of environmental microbes. Similarly, Alkayyali et al. (2021) created Microbe Domestication Pod (MD Pod), a microfluidics platform with chambers and sealed membranes, aiming to load agarose microbeads containing marine bacteria and incubate them *in situ* in marine sediments. Another noteworthy example includes an *in situ* hollow-fiber membrane platform integrated with injectors to maintain the flow of substrates, which was particularly used for continuous fermentation (Aoi et al., 2009). The authors improved the culturability of the cells by up to 12% in comparison to microbial cells inoculated in Petri dishes (Aoi et al., 2009). Lastly, the development of transwell plates by Svenning et al. (2003) allowed for the recovery of methane-oxidizing bacteria by enabling the diffusion of nutrients from the environment to the plate.

Halophilic bacteria and ammonia-oxidizing archaea from coral mucus, for example, have been cultivated using these approaches (Schultz et al., 2022a). Cai et al. (2022) conducted an *in situ* investigation of cold seeps using simple dialysis tubes loaded with a previously cultivated strain of *Erythrobacter flavus*. The authors were able to describe the thiosulfate oxidation pathway for this strain under natural conditions. This study shows how diffusion-based approaches could be used to study microbial metabolism, in addition to improving cultivation methods. This research paves the way for *in situ* screening for exoplanetary biosignatures, where microbes could be grown under natural conditions similar to those occurring on other planets, after which their metabolic traits can be tracked as potential biosignatures. Gevi et al. (2022) used a similar approach to mimic Martian conditions *in vitro* and grow the black fungus *Cryomyces antarcticus*. Metabolomic profiles of the cultures increased the range of possible biosignatures of life on Mars. Variations of *in situ* cultivation techniques include devices designed without membranes, where microbial cells are trapped in micro-wells and fully exposed to the environment (Ingham et al., 2007), as well as membranes with different pore sizes that allow only certain desired microbial cells to pass (Gavrish et al., 2008).

Although these devices vary in application, shape, composition, and size, some of their basic principles are quite similar. Specifically, these instruments often consist of chambers or microchambers where the desired microbial cells are loaded, with membranes separating the inside of the chamber from the external environment. The microbial cells are usually retrieved from an environment of interest and once they are loaded and trapped in the devices, the platforms are set up at the original sampling site. The growth factors naturally occurring in the environment can pass freely through the membranes, allowing the cells to grow (Berdy et al., 2017). This principle could be easily adapted to extreme environments. The optimal device design will depend on the goal. For example, platforms without membranes could be used to cultivate thermophilic, psychrophilic, and piezophilic microbes, since the membrane integrity could be affected by high temperatures, formation of micro-ice crystals, and high hydrostatic pressures, respectively. Even changes in pore sizes caused by dilation could yield uncertain results. All the diffusion-based alternatives discussed herein might be adapted and applied for the recovery of extremophiles from a given environment.

#### 2.2.6. Culturomics

Culturomics is a novel approach that combines next-generation sequencing with high-throughput cultivation by simultaneously using a wide range of culture media with the goal of identifying different compositions that best promote the growth of different microbes in a given sample (Greub, 2012; Lagier et al., 2012). This strategy uses the above-mentioned improvements in cultivation (low-nutrient media, carbon source, variation in incubation, pH, signaling and antibiotic compounds, and co-culture) as well as high-throughput methods [matrix-assisted laser desorption ionization–time of flight mass spectrometry (MALDI-TOF MS) and amplicon sequencing] to retrieve large numbers of microbial colonies (Greub, 2012). Culturomics emerged with the objective of recovering rare and/or new microbial lineages and has led to the cultivation of microbes from different extreme environments that were previously thought to be uninhabitable by microorganisms (Sysoev et al., 2021). Additionally, genomic information from microorganisms and metagenomic data from extreme environments have led to the discovery of habitat-specific genes (Kumar et al., 2017) and provide a basis for the design of specific and unique culture medium conditions based on the metabolic repertoire of target microorganisms to obtain pure cultures (Nielsen et al., 2014). In the near future, metagenome-assisted culturomics will be increasingly valuable for deciphering the community composition of microbial dark matter.

Culturomics has been successfully used to explore human gut microbiota, extending knowledge of human microbial diversity and providing descriptions of new microbial taxa (Lagier et al., 2016; Diakite et al., 2020), but also of extreme environments. For example, by changing the salt concentrations of the growth media, Khelaifia et al. (2018) first described a new halophilic archaeon species, *Haloferax massiliense*, from the human gut. Other new extremophile species with different metabolisms have been successfully retrieved using similar approaches, including thermophiles (Yasir et al., 2019), microbes resistant to reactive oxygen species (Kapinusova et al., 2022), and hyperhalophilies (Durán-Viseras et al., 2021). Using culturomics, Zeng et al. (2021) cultivated and isolated *Gemmatimonas groenlandica* (a representative bacterial species from the phylum Gemmatimonadetes) for the second time. This phylum consists of photoheterotrophic bacteria; however, its members remain largely uncharacterized because their cultivation is uniquely challenging. Sood et al. (2021) recently proposed the use of this approach with some modifications in order to improve the cultivation of extremophiles. The authors suggested applying metagenomics in natural environments as the first step prior to cultivation. Binning metagenome-assembled genomes (MAGs) and single amplified genomes (SAGs) retrieved from a given source could provide insights into the nutritional requirements of the microbes living there. Carbon and nitrogen cycling, as well as the genes related to degradation pathways and adaptations to specific niches, are good traits to search for. Once the screening is complete, culture media could be formulated to meet previously determined requirements (Sood et al., 2021).

#### 2.2.7. Reverse genomics isolation

Reverse genomics isolation is a relatively new approach that enables the culture of hard-to-culture or yet-to-be-cultured microbes, which focuses on using genomic data to guide the cultivation of previously uncultured microorganisms (Cross et al., 2019). This strategy has the potential to support the growth of extremophiles by first applying metagenomics and single-cell genomics (already-available genomes) to obtain genomic data of the microbial community in their natural environment (e.g., nutritional requirements, growth factors, and coculture). Afterward, this knowledge is used to design specific culture conditions that simulate the natural habitat of the microbes, thus enhancing the chances of successfully culturing the target microbe or group of microorganisms (Cross et al., 2019).

Reverse genomics isolation has been successfully implemented to enable the culture of several previously uncultivated microbes, including members of the candidate phyla radiation (CPR) and the Planctomycetes, Verrucomicrobia, and Chlamydia (PVC) superphylum, both of which are considered unknown and enigmatic clusters of microorganisms. Cross et al. (2019) were the first to cultivate and isolate three lineages of previously uncultured Saccharibacteria from a complex community and one human oral SR1 specimen from the human oral cavity. The authors used genome-engineered antibodies as immunofluorescence probes for Saccharibacteria (TM7) as a means to maintain cell viability. Once the cells were sorted, they were cultured using different solid and liquid culture media based on their genomic information. Ibrahim et al. (2022) also used reverse genomics for creating a universal epitope to isolate different members of the Saccharibacteria lineage from the human oral cavity and successfully obtained specific epitopes from species of this superphylum *in silico*.

Overall, these findings highlight the potential of reverse genomics as a promising approach to culture previously uncultivated microbes including but not limited to CPR and PVC members. Additionally, this approach could enable the culture of other uncultured microbes, as well as rare, low abundance, and slow-growing microorganisms to greatly expand our understanding of microbial diversity and functions.

#### 2.2.8. Cell-targeted cultivation

Up to this point, we have mainly discussed expanding the range of extremophile culturability, aiming to recover a wider diversity of microbial isolates than with conventional methods, as well as to discover new taxa. Other approaches such as enrichment can be used when seeking to cultivate certain groups of microorganisms based on metabolic characteristics and/or nutritional requirements. Microorganism screening, however, can focus on taxa of particular taxonomic groups or with specific functional traits depending on the purpose of the study. Cell-targeted cultivation strategies (Cross et al., 2019; Lee et al., 2021) can be roughly divided into two steps: a first trial of cell sorting using different methods, aiming to segregate the target cell and deplete the unwanted ones; followed by integrated cultivation efforts using suitable media. Both steps are challenging because the first requires keeping the cells viable and the second depends on a well-designed cultivation strategy. Moreover, the success of the first step does not guarantee effective culturability.

Alternatively, more feasible methods to retrieve viable environmental microbes include FACS (fluorescence-activated cell sorting; Espina et al., 2022), Live-FISH (fluorescence *in situ* hybridization; Batani et al., 2019), micromanipulators, and laser manipulation systems (optical tweezers and laser microdissection). Additional technical details are provided by Zhang and Liu (2008), Keloth et al. (2018), and Lee et al. (2019). Briefly, optical tweezers and laser microdissection are used in sorting, culturing, and isolating single bacterial cells from a mixed culture by using a microscope to locate, trap, collect, and transfer a single cell to fresh culture media (Huber et al., 1995; Frumkin et al., 2008). Optical tweezers proved a rapid and efficient tool for successfully isolating hyperthermophiles (Huber et al., 1995, 2000) and an extremely halophilic archaeon (Antunes et al., 2008a,b) from a mixed microbial community. Despite being labor-intensive and requiring particular growth conditions, isolation techniques enable the culture and characterization of a variety of undiscovered strains (Alain and Querellou, 2009).

## 3. Bioprospecting the metabolic potential of cultured extremophiles

Environmental parameters such as temperature, pH, salinity, anoxia, pressure, UV and ionizing radiation, and water and nutrient availability can limit microbial life and affect the structure and diversity of microbial communities (Paulino-Lima et al., 2013; Shock and Boyd, 2015; Merino et al., 2019). Many natural terrestrial and aquatic environments (e.g., deserts, polar regions, geothermal environments, supersaturated salt pools, acidic or soda lakes, and the deep sea) were originally thought to be too harsh to harbor life (Carré et al., 2022). However, more recent studies have demonstrated the occurrence of a wide diversity of organisms in these extreme habitats (Jurelevicius et al., 2012; Cury et al., 2015; Lopez et al., 2019; Shu and Huang, 2022). Microorganisms able to tolerate, survive, or require extreme conditions to grow optimally are known as extremophiles (Brock and Freeze, 1969; MacElroy, 1974; Rothschild and Mancinelli, 2001), whereas those that grow optimally under multiple extreme stress conditions are known as polyextremophiles (Capece et al., 2013). To thrive in such extreme conditions, extremophiles must develop a range of adaptations that provide a unique perspective on the fundamental characteristics of biological processes, as well as the broad metabolic diversity and physiological capabilities of these microorganisms (Canganella and Wiegel, 2014; Salwan and Sharma, 2020). Although knowledge of extremophile biology has progressed considerably in recent decades, (poly)extremophiles remain largely unexplored mainly due to the difficulty of culturing these microorganisms in a laboratory setting. Here, we highlight the importance of cultured extremophilic microorganisms and briefly discuss their potential for biotechnology and space-related sciences.

### 3.1. Biotechnological applications of extremophilic microbes

Extremophiles may generate bioproducts with important biotechnological applications (Bull and Goodfellow, 2019) for use in many areas of biotechnology, including agriculture, medicine, and the petroleum and pharmaceutical industries (Reis-Mansur et al., 2019; Schultz and Rosado, 2019, 2020; Zhu et al., 2020; Schultz et al., 2022b). One noteworthy example is the global enzyme market, which was valued at US$6.4 billion in 2021 (BCC, 2022). However, the commercial enzyme market has struggled to meet the increasing requirements of the industrial sectors (i.e., textile manufacturing, pulp and paper, biofuel), mainly because most enzymes currently available in the market are only active within a very narrow range of conditions because they originate from mesophilic organisms. Therefore, they quickly lose activity under the extreme conditions found in industrial processes (temperature, pH, pressure, solvent concentrations, and ion concentration). Enzymes from polyextremophiles hold the promise of fulfilling industrial needs (Kara and Liese, 2019; Sysoev et al., 2021) and may produce robust enzymes with higher activity and stability under the extreme conditions that characterize industrial processes (Rampelotto, 2016; Mesbah, 2022). From samples obtained in Antarctica, Monsalves et al. (2020) isolated a psychrotolerant and UV-C resistant bacterium that produces catalase, an enzyme commonly used in the textile industry, that was thermoactive and thermostable under high temperatures (optimal activity at 50°C at pH 7.0). Sheikhi et al. (2012) screened microorganisms from geothermal sites for thermoalkaliphilic laccase, an important enzyme used in the biofuel industry. Using a sample from a polar volcano, Márquez and Blamey (2019) isolated thermophilic microorganisms capable of producing thermophilic amine-transaminase, an enzyme with important pharmacological applications.

Extremophiles are also promising sources of biosurfactants, a versatile type of chemical that is widely used in the oil, pharmaceutical, and food industries, which generates billions of dollars per year (Cameotra et al., 2010; Schultz and Rosado, 2020; da Silva et al., 2022; Schultz et al., 2022b). Currently, most commercially available surfactants are derived from petroleum products (Jemil et al., 2016). However, replacing chemically synthesized compounds with compounds of biological origin may have advantages (Banat et al., 2014; Perfumo et al., 2018), such as low toxicity, high biodegradability, production from renewable materials, and capability to remain active and stable under extreme temperatures, pH, and salinity (Vijayakumar and Saravanan, 2015; Jemil et al., 2016; Schultz et al., 2022b). For example, Schultz et al. (2022b) screened for oil-degrading and biosurfactant-producing thermophiles by investigating the thermophilic microbial community of Antarctic geothermal sites for applications in the bioremediation of oil-contaminated sites and microbial-enhanced oil recovery processes. The authors reported that the majority of the isolated microbes were able to grow in a culture medium supplemented with crude oil as the only carbon source and exhibited excellent performance in terms of biosurfactant production and emulsification stability at 100°C. Additionally, extremophiles require a large suite of specialized metabolites to overcome their hostile environments and thus represent a potentially valuable reservoir of novel bioactive molecules. However, the secondary metabolites derived from microbiomes thriving in extreme habitats have remained largely unexplored, and thus represent new frontiers in drug discovery (Sayed et al., 2020). Therefore, microbes from extreme environments are a uniquely promising source of novel potent anticancer molecules, as reported by Sagar et al. (2013a,b) and Esau et al. (2019), who screened samples from Red Sea brine pools, in addition to other diseases such as Alzheimer’s disease, Parkinson’s disease, hypercholesterolemia, and epilepsy. Biomolecules from extremophiles are also valuable for other medical applications such as the production of halocins, diketopiperazines, DNA polymerase, and lipases (Kumar et al., 2017), as well as carotenoids, which are important for the production of antibiotics and cosmetics related to UV protection and antioxidant agents (Inoue et al., 2017). Additionally, these compounds have important implications for agriculture, as microbial bioactive compounds can be used to achieve high crop yields, mitigate pathogens (Rachid et al., 2015; Rao et al., 2022), and promote soil health (Yadav, 2021).

### 3.2. Extremophiles on Earth and beyond: implications in astrobiology

In addition to the biotechnological applications of extremophilic microbes or their products, extreme microbes may provide significant insights for astrobiology research due to their capacity to survive in conditions analogous to or simulated extraterrestrial habitats or conditions (Lage et al., 2012). Considering that temperature, radiation, gravity, and salinity are the primary parameters that determine life in outer space, the study of survival strategies in extreme conditions helps to define questions regarding planetary habitability and to identify possible targets for the search for extraterrestrial life (Rothschild and Mancinelli, 2001; Bonch-Osmolovskaya, 2010).

Most ecosystems inhabited by extremophiles on Earth resemble planetary bodies in outer space (Schmid et al., 2020). Microorganisms isolated from these extreme habitats considered analogous to extraterrestrial environments are highly adapted and are good candidates for astrobiological studies [e.g., astrobiological models, adaptation mechanisms, strategies underlying survival in extreme conditions, and potential (novel) biosignatures that can be used in habitable zones beyond Earth; Lopez et al., 2019; Jebbar et al., 2020], yielding potential clues as to whether (and how) life may exist and persist on other planetary bodies (Lopez et al., 2019; Coleine and Delgado-Baquerizo, 2022), and even provide tools to guide future colonization, if this is desired (Lopez et al., 2019). So far, prokaryotes are the most-studied models for astrobiology (Seyler et al., 2020; Abbott and Pearce, 2021), including members of the (i) halophilic archaea (class Haloarchaea), which are typically used for Mars studies, due to their ability to withstand salinity and perchlorates, anaerobic conditions, high levels of UV and ionizing radiation, subzero temperatures, desiccation, and toxic ions (Stan-Lotter and Fendrihan, 2015; DasSarma and DasSarma, 2017); (ii) *Deinococcus radiodurans*, a bacterium that is widely known for its high resistance to radiation (up to 10,000 Gy; Daly, 2011) and its importance in the context of planetary protection and panspermia (Kawaguchi et al., 2013); and (iii) representatives of the genus *Bacillus*, which have repeatedly demonstrated their ability to survive in many extreme conditions encountered in outer space. These members include (i) *Bacillus subtilis*, which is known to survive in conditions similar to those on Mars (extreme dryness, high radiation levels, and high concentrations of perchlorate salts) (Nicholson et al., 2000), and (ii) *Bacillus pumilus*, a model organism that is currently used to assess the habitability of Europa (icy moon of Jupiter) based on its capability to survive in extreme temperatures, low nutrient availability, dryness, and UV-C radiation (Stepanov et al., 2016). Photosynthetic extremophilic organisms and fungi are also reported as good models. Members of the order Chroococcidiopsidales (e.g., *Chroococcidiopsis*) have proved to be interesting candidates for Mars and icy moons, tolerating many years of desiccation, high doses of ionizing radiation, microgravity, high concentrations of perchlorates, and low temperatures (Cosciotti et al., 2019; Billi et al., 2021; Napoli et al., 2022). Additionally, strains of *Cladosporium sphaerospermum* and *Cremonium murorum,* both isolated from a reactor at the Chernobyl Nuclear Plant, are able to extract energy from ionizing radiation, making them viable models of life in space, with its constant cosmic radiation (Blachowicz et al., 2019).

Experiments in the stratosphere and in laboratory-based simulations can facilitate future *in situ* planetary explorations. For instance, fungi, yeasts, bacteria, and cyanobacteria isolated from terrestrial and space-related environments like the International Space Station (ISS) have survived in the stratosphere under multiple Mars-like conditions, such as cold, dry, oligotrophic environments, intense UV radiation, and low pressure (Blachowicz et al., 2019; Cortesão et al., 2019). Additionally, several missions have used balloons to transport biological samples to the high-elevation environment of the stratosphere (Smith et al., 2014; DasSarma et al., 2020). Pulschen et al. (2018) investigated cold-adapted UV-resistant yeasts (black-pigmented *Exophiala* sp. and non-pigment-producing *Naganishia friedmannii*) isolated from a volcano in the Atacama Desert, and both microorganisms showed higher survival rates than *Bacillus subtilis* spores, thus confirming the remarkable resilience of yeast and making them good candidate models for further research. In a recent study, fungal spores (*Aspergillus niger*) and bacterial cells (*Salinisphaera shabanensis, Staphylococcus capitis,* and *Buttiauxella* sp.) were launched on a NASA scientific balloon flight into the stratosphere (Cortesão et al., 2019), and the authors concluded that the halophilic bacterium *S. shabanensis* and spores of the pigmented fungus *A. niger* were the most resistant microbes, with a 2- and 4-log reduction. However, additional efforts are needed to further develop ground-based systems such as laboratory-based simulation chambers to mimic the conditions in outer space, (exo)planets, and moons to determine the capacity of terrestrial microorganisms to survive in extraterrestrial conditions, including microgravity, UV radiation, gas composition, temperature, pressure, and humidity. Among the culturable microorganisms, extremophiles have been extensively placed in simulated conditions, such as the ability of the thermophiles *Sulfolobus solfataricus, Haloterrigena hispanica, Thermotoga neapolitana,* and *Geobacillus thermantarcticus* to survive under Mars-like conditions (Mastascusa et al., 2014). All strains, particularly *S. solfataricus*, proved highly resistant, continuing to grow after exposure to temperature variation, with a slight effect on growth from UV radiation.

## 4. Final remarks

Extremophile research has consistently remained at the cutting edge of microbiology, opening unexplored territory in our understanding of life and its limits. After more than five decades of research focused on exploiting extreme environments to unveil extremophile taxonomy, functions, and applicability in society, microbes capable of thriving in multiple harsh conditions have garnered increasing attention among the scientific community, particularly in the biotechnology sector and, more recently, in astrobiology and space-related studies. The more we learn about extremophiles, the more intriguing they become, opening broad questions regarding their evolution, adaptations, and diversity. So far, most attempts to characterize and describe the extremosphere have involved conventional culture methods. However, extremophiles commonly fail to grow under standard laboratory conditions and remain uncharacterized. New cultivation approaches are thus essential to enable the recovery of the still-uncultured fraction of the microbial dark matter, thus improving culturability rates and enabling new discoveries.

Recent years have seen extensive advances in high-throughput cultivation, techniques to mimic the natural environment, and the development of a variety of devices to cultivate yet-to-be-cultured microbes. Culturomics coupled with focused genome-guided cultivation efforts and *in situ* cultivation by using materials modified according to each extreme environment are promising strategies for the characterization of extreme microbiomes. These innovative strategies complement the current cultivation-independent approaches, such as metagenomics and metatranscriptomics, to further understand the physiology of extremophiles. Thus, culturomics is an important approach that has enabled scientists to extend our knowledge about extremophiles, including the description of new species and the discovery of new metabolic pathways with high biotechnological potential. However, the application of this strategy for extremophile research is still in its infancy. In this review, we summarized the current strategies used to culture the environmental microbial diversity, including the extremosphere. We hope that the topics discussed herein will aid microbiologists in untangling the composition and functions of extremophilic microbiomes, describing novel taxa, and screening microorganisms for new biotechnological applications.

## Author contributions

JS, FM, RP, and ASR: investigation and writing—original draft, conceptualization, review, and editing. ASR: funding acquisition and supervision. All authors contributed to the article and approved the submitted version.

## Funding

This study was supported by a KAUST Baseline Grant (BAS/1/1096-01-01) to ASR. FM received support from the National Council for Scientific and Technological Development (CNPq).

## Conflict of interest

The authors declare that the research was conducted in the absence of any commercial or financial relationships that could be construed as a potential conflict of interest.

## Publisher’s note

All claims expressed in this article are solely those of the authors and do not necessarily represent those of their affiliated organizations, or those of the publisher, the editors and the reviewers. Any product that may be evaluated in this article, or claim that may be made by its manufacturer, is not guaranteed or endorsed by the publisher.
